# Supplementary material for: Charge symmetry breaking in neutral polyzwitterions
Source: Nat Commun. 2025 Apr 13;16:3507. doi: 10.1038/s41467-025-58928-7 (PMC11994810; doi:10.1038/s41467-025-58928-7)
Supplement: Supplementary file 1 — Supplementary Information [file 41467_2025_58928_MOESM1_ESM.pdf]

Supplementary Information

Charge Symmetry Breaking in Neutral Polyzwitterions

Yeseul Lee[1] & Murugappan Muthukumar[1,*]

[1]Department of Polymer Science and Engineering,

University of Massachusetts, Amherst, Massachusetts 01003, USA

*email: muthu2346@gmail.com

1 Supplementary Figures

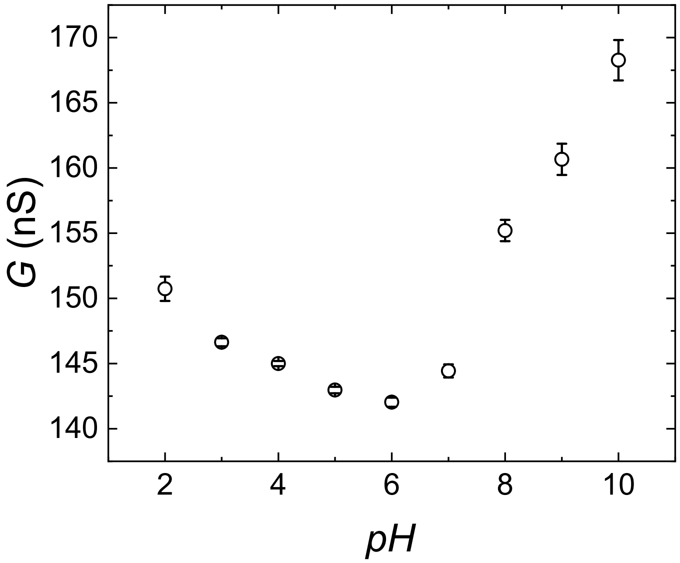

Supplementary Fig. 1: Pore conductance measurement at different pH values in 1 M KCl. The isoelectric point is the pH value where the pore conductance is minimum [1] The isoelectric point of pH 6 here is consistent with literature value [2]. The error bars show the standard deviations of pore conductance values from three measurements at one pH value. The pore diameter was 21 nm, and the voltage range was −0.5 V to +0.5 V.

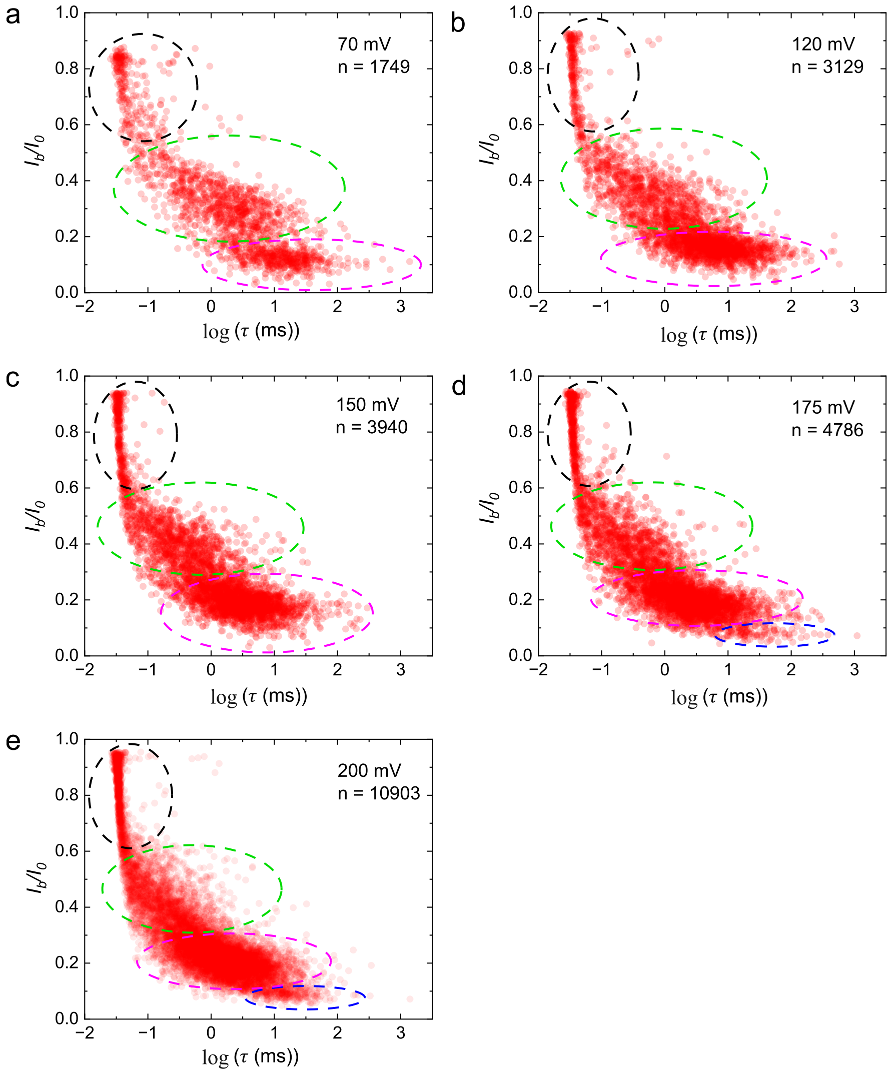

Supplementary Fig. 2: Distributions of blockage events and blockage durations for 100 nM PSBMA in 1 M KCl, 10 mM HEPES, pH 7. (a-e) Event scatter plots at different voltages. Black, green, magenta, and blue ellipses correspond to unsuccessful translocations (collisions), single-file translocations, intra-pore-structured translocations, and intra-pore-double-structured translocations, respectively. Pore diameter: 3.7 nm, sampling rate: 250 kHz, low-pass filter frequency: 100 kHz, Gaussian filter frequency: 10 kHz.

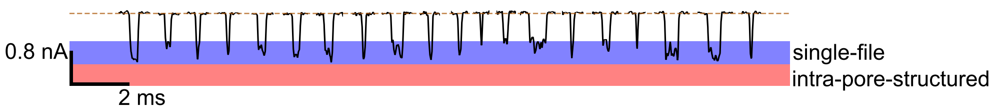

Supplementary Fig. 3: Current traces of moderate blockages (single-file translocations) for 100 nM PSBMA in 1 M KCl, 10 mM HEPES, pH 7 at 175 mV. Pore diameter: 3.7 nm, sampling rate: 250 kHz, low-pass filter frequency: 100 kHz, Gaussian filter frequency: 10 kHz.

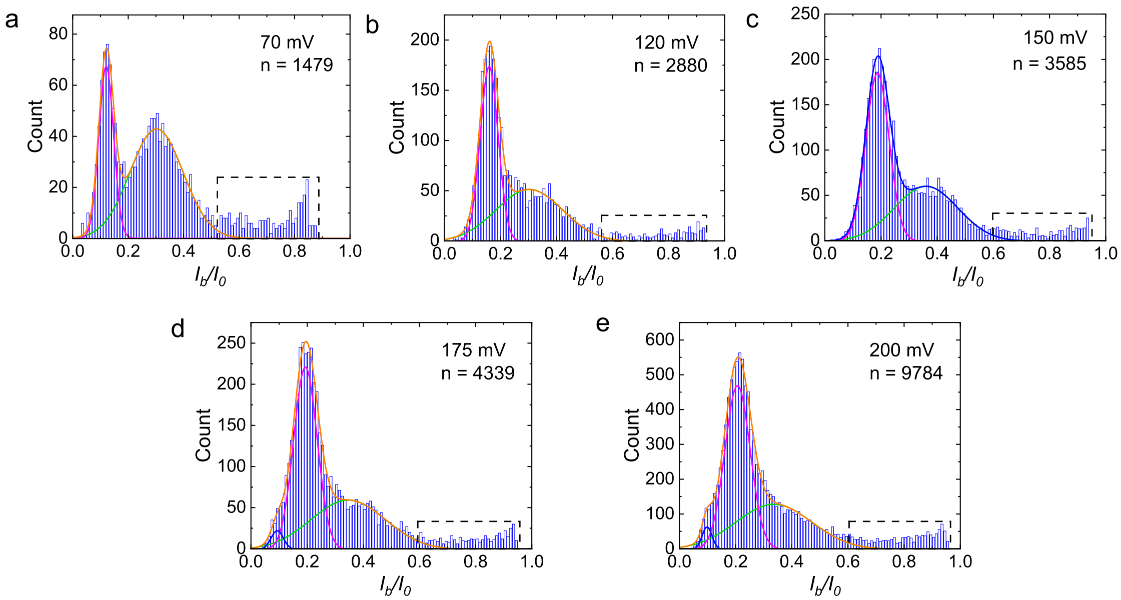

Supplementary Fig. 4: Histograms of I_b/I_0 for 100 nM PSBMA in 1 M KCl, 10 mM HEPES, pH 7 at different voltages: (a) 70 mV (n=1479), (b) 120 mV (n=2880), (c) 150 mV (n=3585), (d) 175 mV (n=4339), and (e) 200 mV (n= 9784). The black boxes denote unsuccessful translocations (collisions); green, magenta, and blue curves correspond to single-file, intra-pore-structured, and intra-pore-double-structured translocations, respectively. Orange curves are envelopes of single-file, intra-pore-structured, and intra-pore-double-structured translocations. Pore diameter: 3.7 nm, sampling rate: 250 kHz, low-pass filter frequency: 100 kHz, Gaussian filter frequency: 10 kHz.

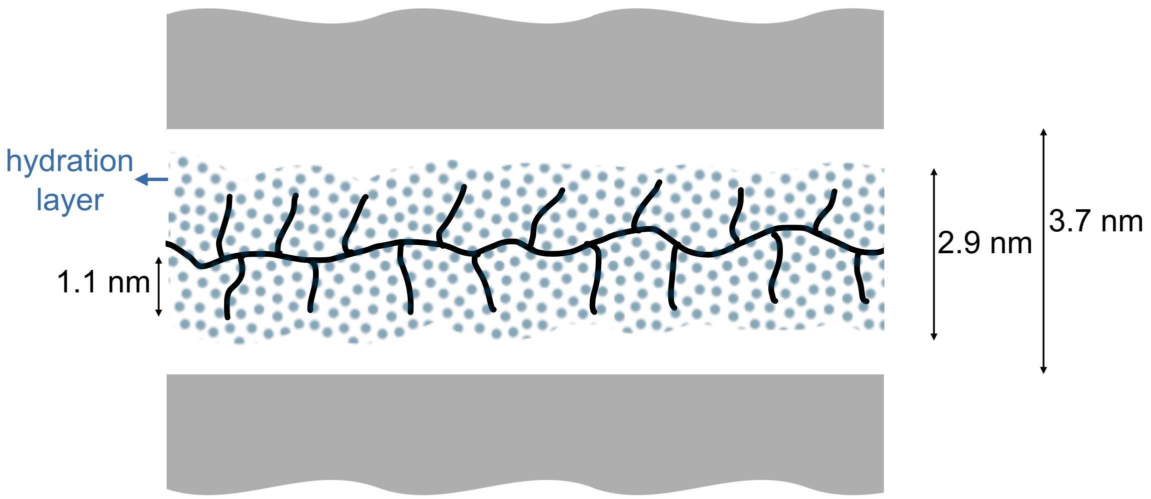

Supplementary Fig. 5: Cartoon of conformation of PSBMA chain under single-file translocation. Minor fluctuations of the backbone and hydration layer along the polymer make the effective cross-sectional diameter of ∼2.9 nm.

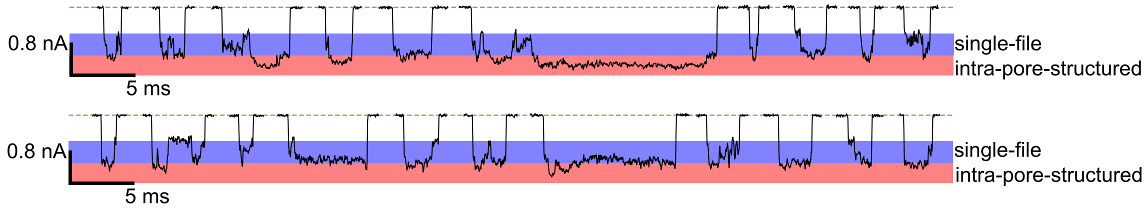

Supplementary Fig. 6: Current traces of deep blockages (intra-pore-structured translocations) for 100 nM PSBMA in 1 M KCl, 10 mM HEPES, pH 7 at 175 mV. Pore diameter: 3.7 nm, sampling rate: 250 kHz, low-pass filter frequency: 100 kHz, Gaussian filter frequency: 10 kHz.

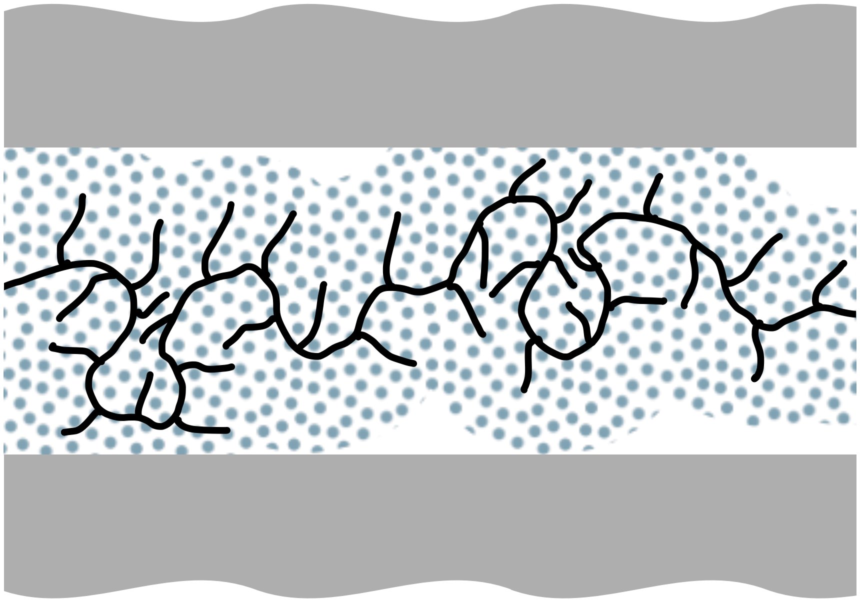

Supplementary Fig. 7: Cartoon of conformation of PSBMA chain under intra-pore-structured translocation.

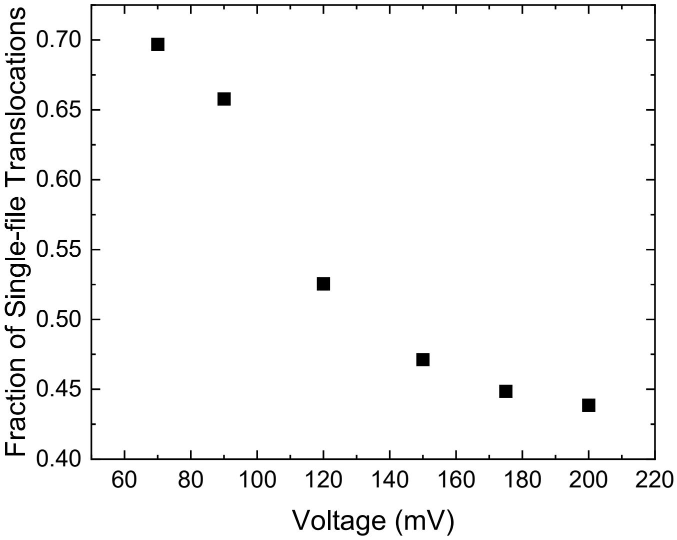

Supplementary Fig. 8: Voltage dependence of fraction of single-file translocations for 100 nM PSBMA in 1 M KCl, 10 mM HEPES, pH 7. The fractions are obtained from multiple Gaussian distribution fits to I_b/I_0 histograms. Pore diameter: 3.7 nm, sampling rate: 250 kHz, low-pass filter frequency: 100 kHz, Gaussian filter frequency: 10 kHz.

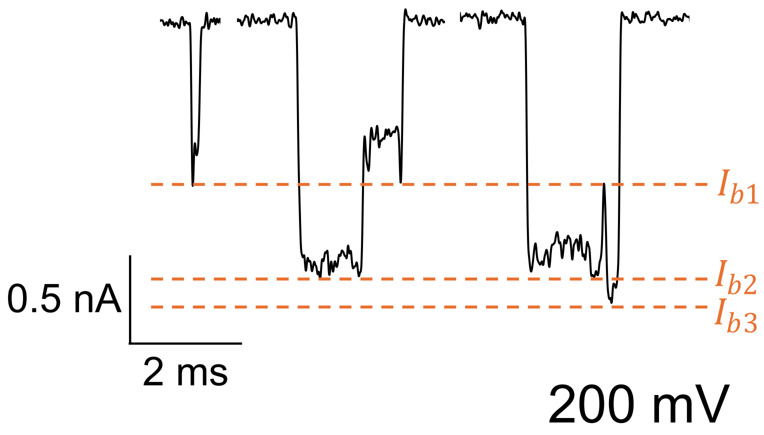

Supplementary Fig. 9: Current traces of single-file translocation, intra-pore-structred translocation, and intra-pore-double-structured translocation for 100 nM PSBMA in 1 M KCl, 10 mM HEPES, pH 7 at 200 mV. Pore diameter: 3.7 nm, sampling rate: 250 kHz, low-pass filter frequency: 100 kHz, Gaussian filter frequency: 10 kHz.

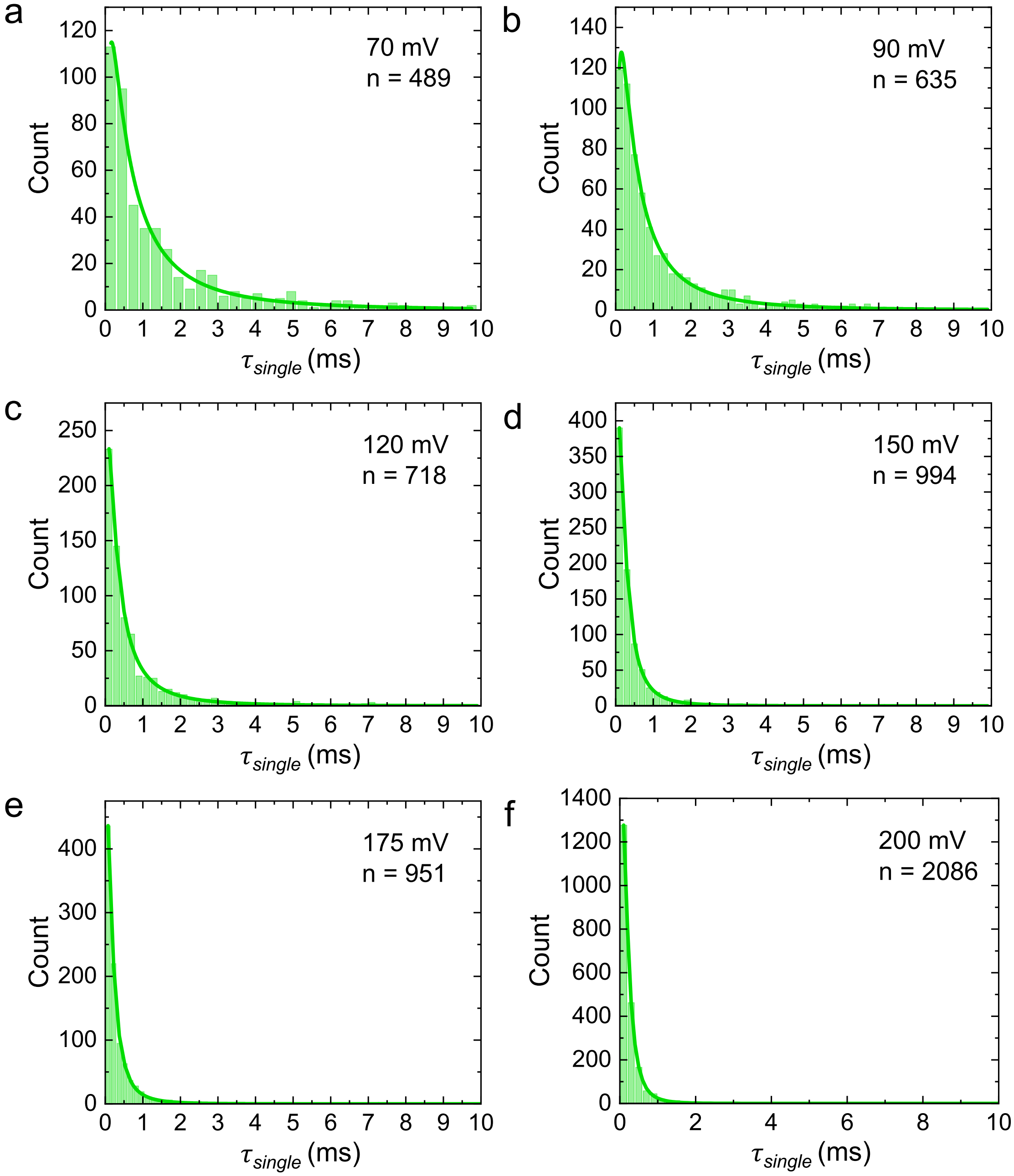

Supplementary Fig. 10: Histograms of single-file translocation times for 100 nM PSBMA in 1 M KCl, 10 mM HEPES, pH 7 at different voltages: (a) 70 mV (n=489), (b) 90 mV (n=635), (c) 120 mV (n=718), (d) 150 mV (n= 994), (e) 175 mV (n=951), and (f) 200 mV (n=2086). Solid curves are log-normal fits. Pore diameter: 3.7 nm, sampling rate: 250 kHz, low-pass filter frequency: 100 kHz, Gaussian filter frequency: 10 kHz.

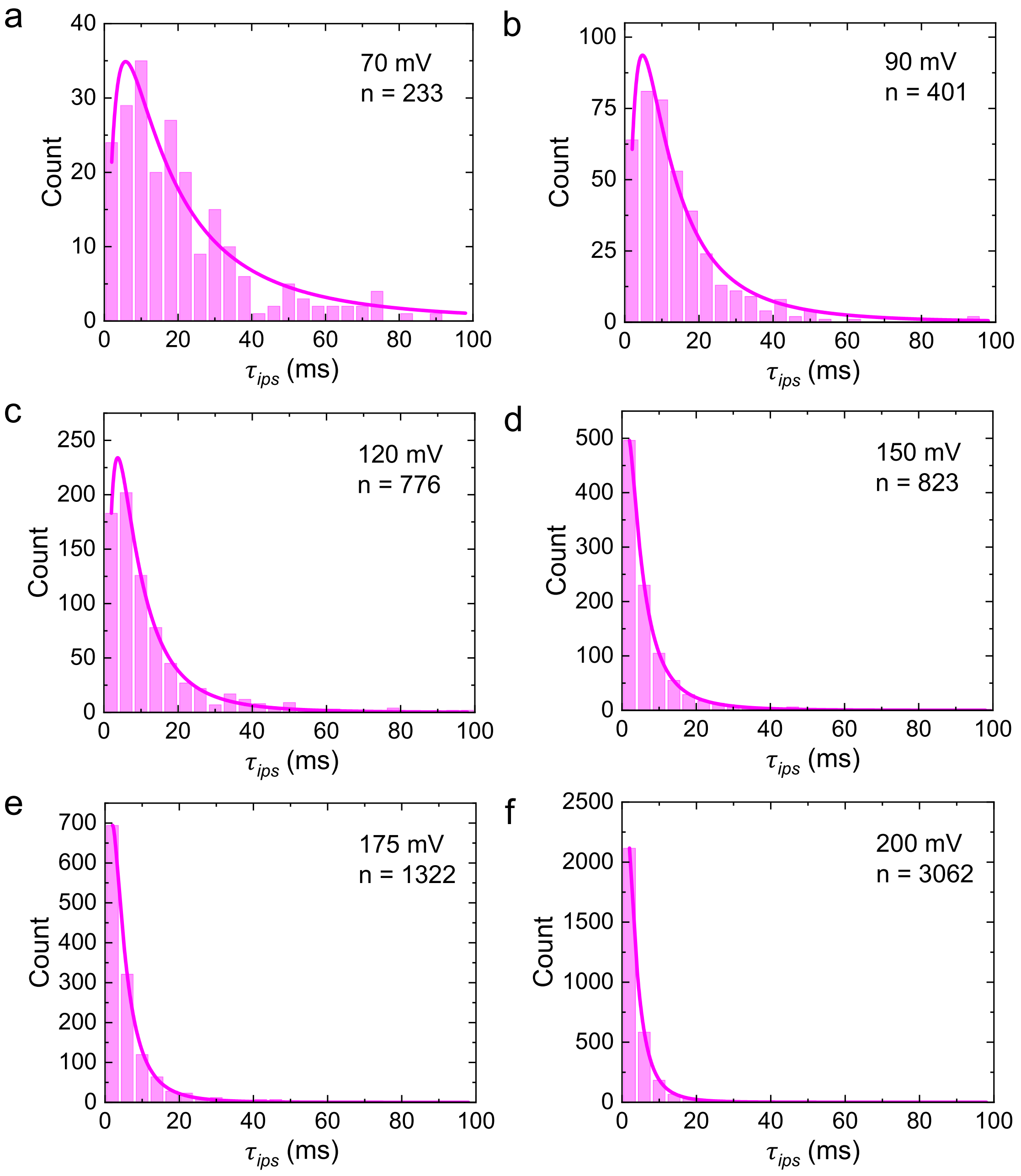

Supplementary Fig. 11: Histograms of intra-pore-structured translocation times for 100 nM PSBMA in 1 M KCl, 10 mM HEPES, pH 7 at different voltages: (a) 70 mV (n=233), (b) 90 mV (n=401), (c) 120 mV (n=776), (d) 150 mV (n=823), (e) 175 mV (n=1322), and (f) 200 mV (n=3062). Solid curves are log-normal fits. Pore diameter: 3.7 nm, sampling rate: 250 kHz, low-pass filter frequency: 100 kHz, Gaussian filter frequency: 10 kHz.

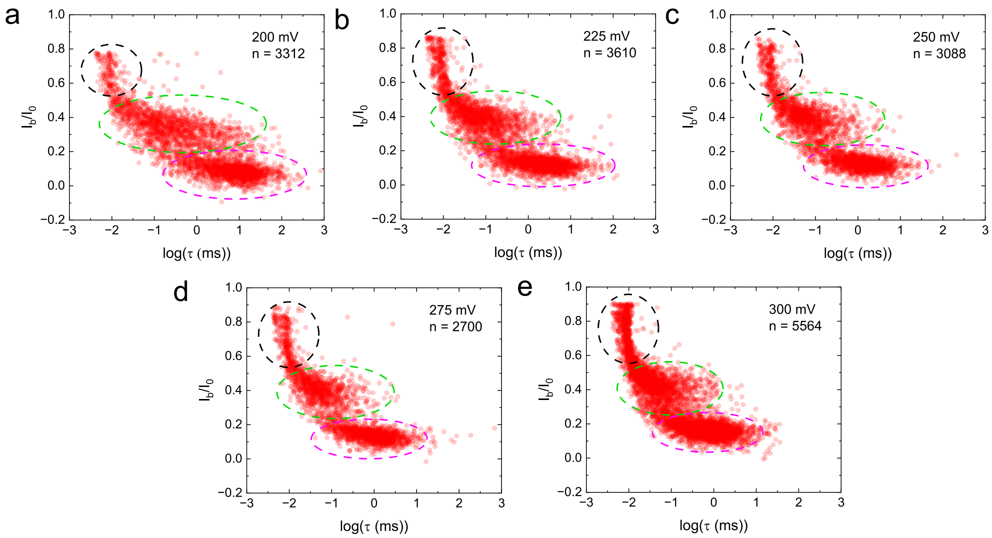

Supplementary Fig. 12: Event scatter plots for 100 nM PSBMA in 3.6 M LiCl, 10 mM HEPES, pH 6 at different voltages: (a) 200 mV (n=3312), (b) 225 mV (n=3610), (c) 250 mV (n=3088), (d) 275 mV (n=2700), and (e) 300 mV (n=5564). Black, green, and magenta ellipses correspond to unsuccessful translocations (collisions), single-file translocations, and intra-pore-structured translocations, respectively. Pore diameter: 3.7 nm, sampling rate: 250 kHz, low-pass filter frequency: 100 kHz.

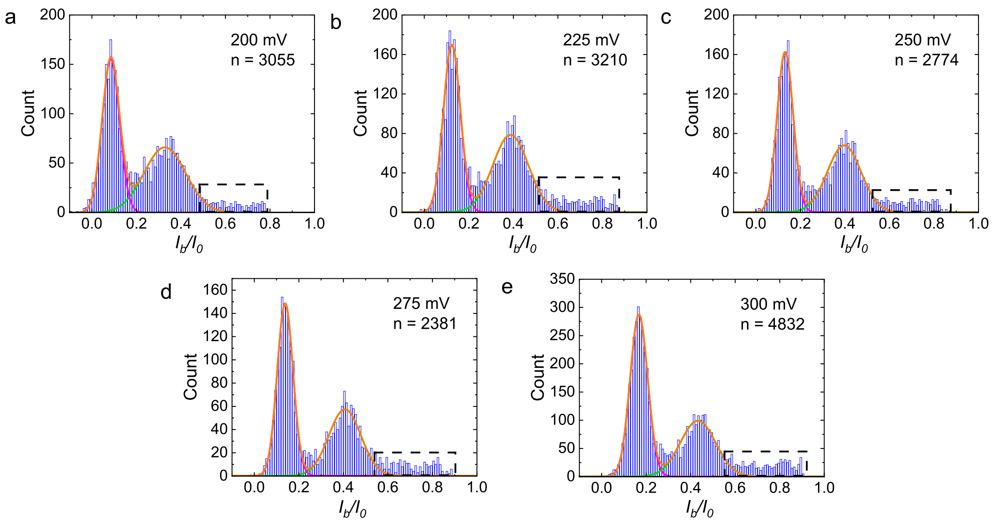

Supplementary Fig. 13: Histograms of I_b/I_0 for PSBMA in 3.6 M LiCl, 10 mM HEPES, pH 6 at different voltages: (a) 200 mV (n=3055), (b) 225 mV (n=3210), (c) 250 mV (n=2774), (d) 275 mV (n=2381), and (e) 300 mV (n=4832). The black boxes denote unsuccessful translocations (collisions); green and magenta curves correspond to single-file and intra-pore-structured translocations, respectively. Orange curves are envelopes of single-file and intra-pore-structured translocations. Pore diameter: 3.7 nm, sampling rate: 250 kHz, low-pass filter frequency: 100 kHz.

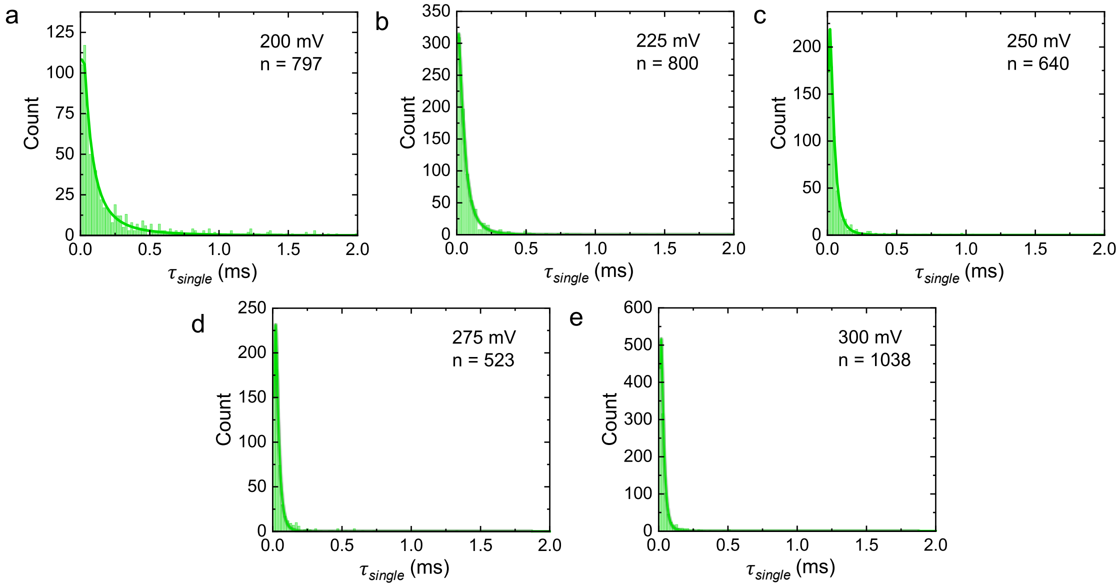

Supplementary Fig. 14: Histograms of single-file translocation times for PSBMA in 3.6 M LiCl, 10 mM HEPES, pH 6 at different voltages: (a) 200 mV (n=797), (b) 225 mV (n=800), (c) 250 mV (n=640), (d) 275 mV (n=523), and (e) 300 mV (n=1038). Solid curves are log-normal fits. Pore diameter: 3.7 nm, sampling rate: 250 kHz, low-pass filter frequency: 100 kHz.

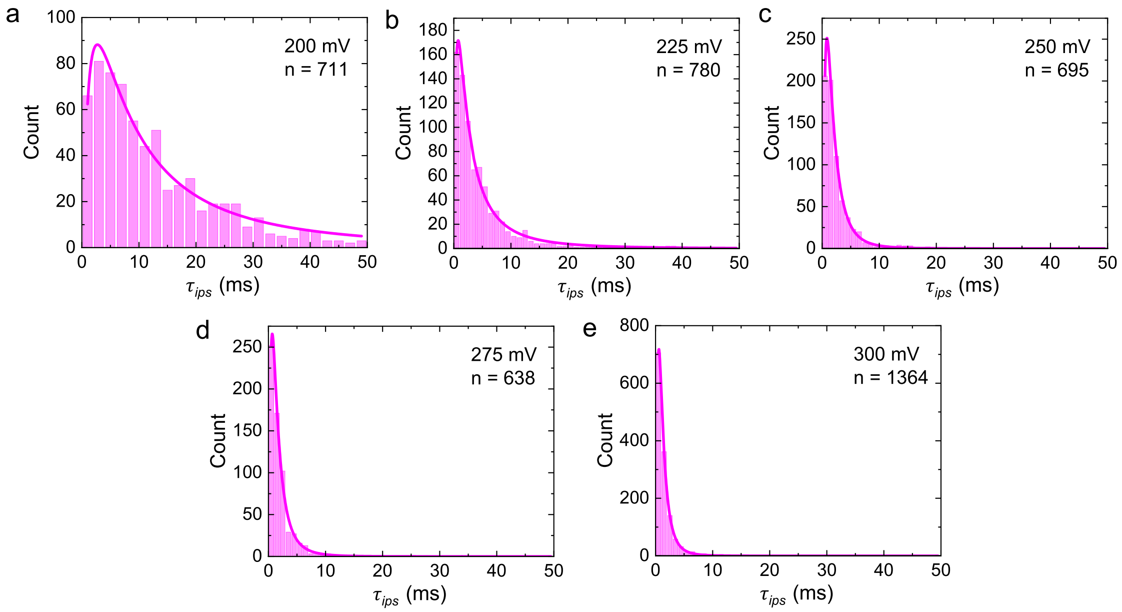

Supplementary Fig. 15: Histograms of intra-pore-structured translocation times for PSBMA in 3.6 M LiCl, 10 mM HEPES, pH 6 at different voltages: (a) 200 mV (n=711), (b) 225 mV (n=780), (c) 250 mV (n=695), (d) 275 mV (n=638), and (e) 300 mV (n=1364). Solid curves are log-normal fits. Pore diameter: 3.7 nm, sampling rate: 250 kHz, low-pass filter frequency: 100 kHz.

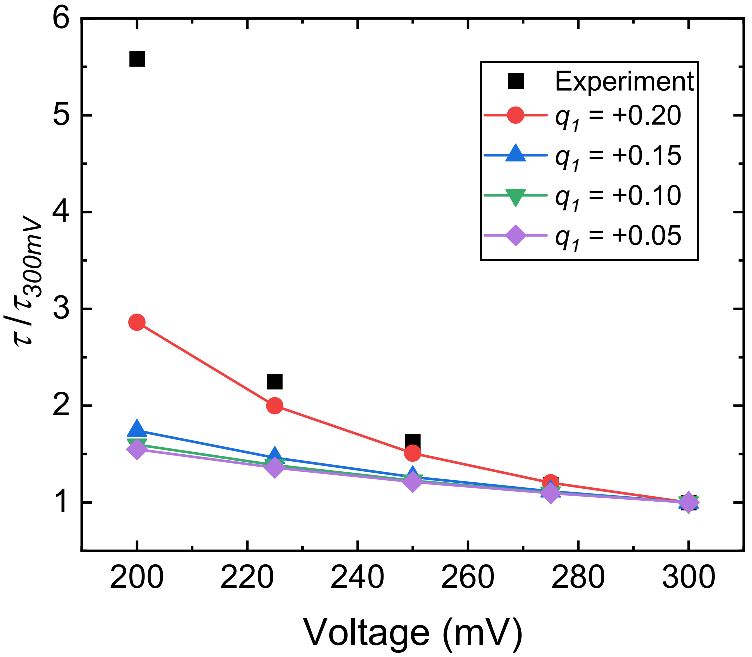

Supplementary Fig. 16: Fitting experimental data for PSBMA translocation in 3.6 M LiCl, 10 mM HEPES, pH 6 in Figure 5d to theoretical values. q_2 was set as -0.25.

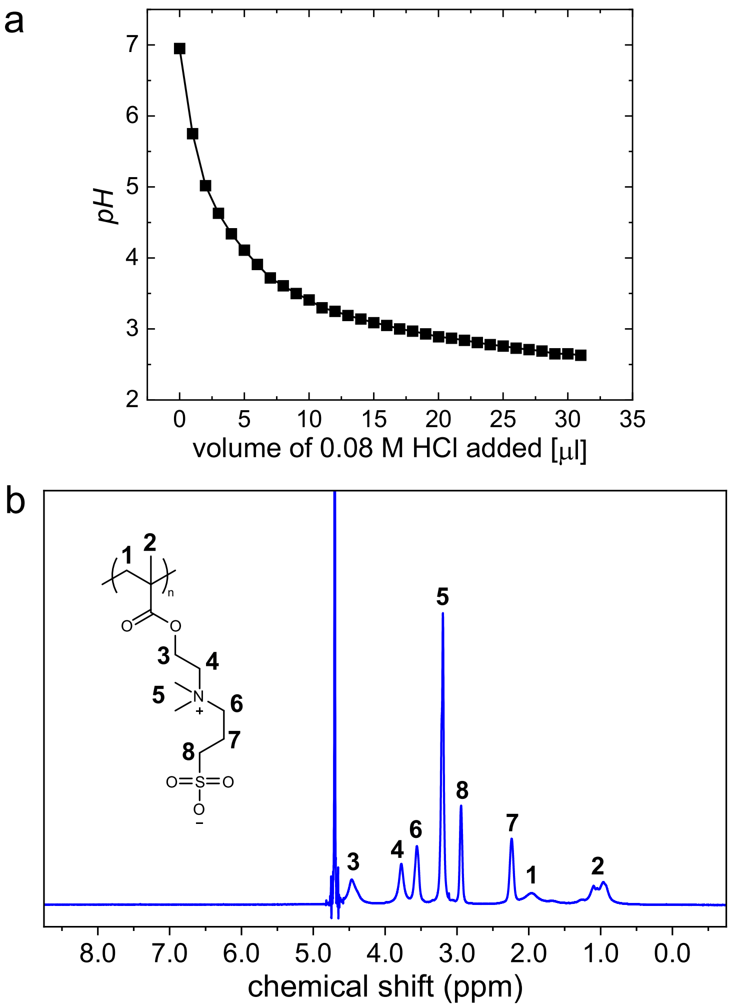

Supplementary Fig. 17: Absence of hydrolysis of SBMA monomers. (a) pH titration curve of 1 mL of 80 μM PSBMA in 1 M KCl titrated with 0.08 M HCl. 1 μl of titrant was added at each data point. (b) ^{1}H NMR spectrum of PSBMA in D_2O with 1 M sodium chloride. ^{1}H NMR (500 MHz, 1 M NaCl/D_2O, δ): 4.57-4.30 (2H), 3.87-3.68 (2H), 3.65-3.49 (2H), 3.30-3.10 (6H), 3.00-2.87 (2H), 2.34-2.13 (2H), 2.13-0.74 (5H + CTA).

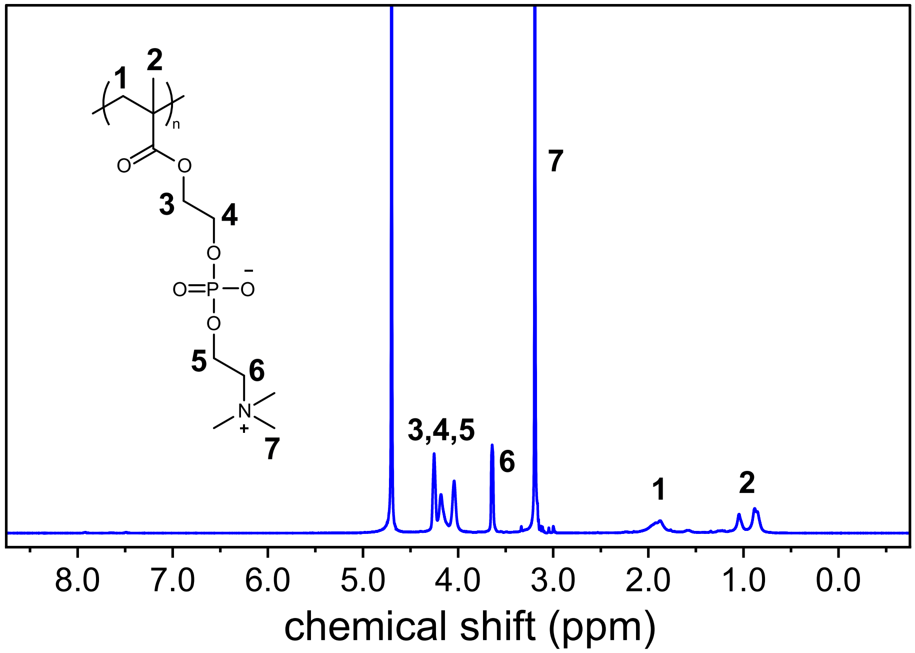

Supplementary Fig. 18: ^{1}H NMR spectrum of PMPC in 0.1 M NaCl and D_2O. ^{1}H NMR (500 MHz, 0.1 M NaCl/D_2O, δ): 7.96-7.44 (CTA), 4.30-3.95 (6H), 3.64 (2H), 3.19 (9H), 2.14-0.75 (5H + CTA).

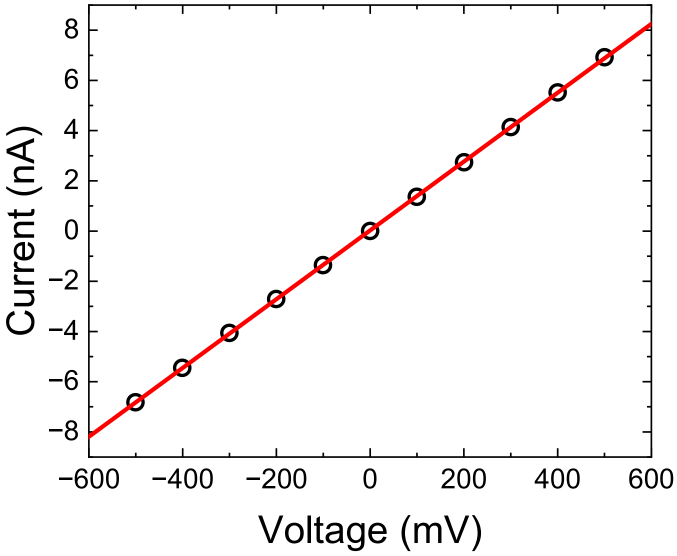

Supplementary Fig. 19: An example of I-V curve after pore fabrication. The current was measured in conditioning buffer. The red line is a linear fit. A pore was used only when its I-V curve was ohmic.

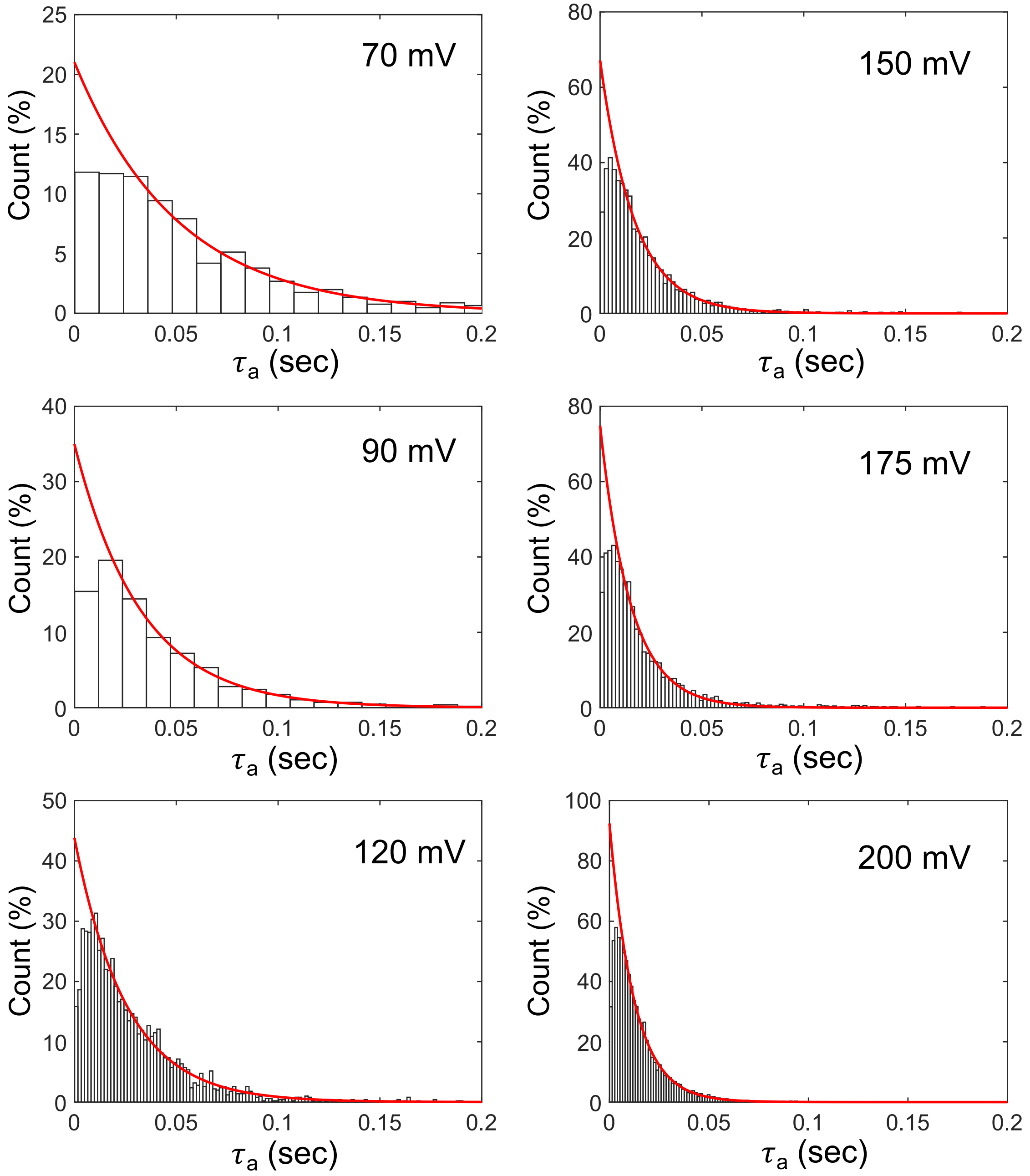

Supplementary Fig. 20: Histograms of τ_a for 100 nM PSBMA in 1 M KCl, 10 mM HEPES, pH 7 at different voltages. The curves in red are exponential fits. Pore diameter: 3.7 nm, sampling rate: 250 kHz, low-pass filter frequency: 100 kHz, Gaussian filter frequency: 10 kHz.

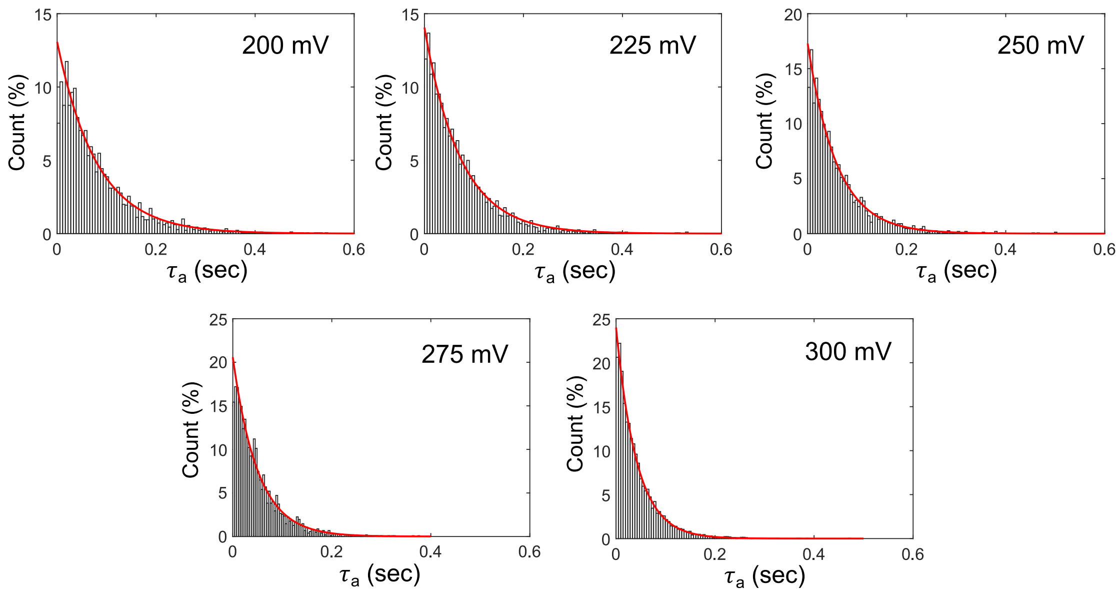

Supplementary Fig. 21: Histograms of τ_a for 100 nM PSBMA in 3.6 M LiCl, 10 mM HEPES, pH 6 at different voltages. The curves in red are exponential fits. Pore diameter: 3.7 nm, sampling rate: 250 kHz, low-pass filter frequency: 100 kHz.

2 Supplementary Tables

Supplementary Table 1: Contributions from charge-electric field interaction F_{pE}/k_BT and chain entropy F_{ent}/k_BT to the free energy in units of k_BT at each stage of polyzwitterion translocation. γ, z, and z_p are defined in Supplementary Note 2.

Stage	F_{pE}/k_BT	F_{ent}/k_BT
1	$\frac{v}{M}(q\frac{m^2}{2} - p'm)$	$-(N-m)\ln z + (1-\gamma)\ln(N-m) - m\ln z_p$
2	$v(q\frac{M}{2} - p')$	$-(N-M)\ln z + (1-\gamma)\ln(N-m)(m-M) - M\ln z_p$
3	$\frac{v}{M}[q\frac{M^2-(m-N)^2}{2} - p'(M+N-m)]$	$-(m-M)\ln z + (1-\gamma)\ln(m-M) - (M+N-m)\ln z_p$

Supplementary Table 2: Contributions from pore wall-chain interaction F_{pore}/k_BT and free energy in receiver chamber F_{bE}/k_BT to the free energy in units of k_BT at each stage of polyzwitterion translocation. ϵ_0 and q_b are defined in Supplementary Note 2.

Stage	F_{pore}/k_BT	F_{bE}/k_BT
1	$\epsilon_0 m$	0
2	$\epsilon_0 M$	$q_b v(m-M)$
3	$\epsilon_0(M+N-m)$	$q_b v(m-M)$

3 Supplementary Notes

Supplementary Note 1

Interpretation of Deep Blockages: There are two possible explanations for the deep blockades. In the first explanation, the chain conformation inside the nanopore can adopt a short hairpin-like kink which then proceeds to complete the translocation process. The formation of such kinks is not prohibitive, because the cross-sectional diameter of the polymer is comparable to the pore radius and there are always conformational fluctuations at the experimental conditions. The kink formation does not necessarily have to occur from the start of the pore entrance but can occur at any location inside the nanopore. This is consistent with the experimental observation that the intra-pore-structured translocations do not always begin with a deeper blockade, as desribed in the next paragph. Since it is unlikely that two molecules simultaneously move through the nanopore given the cross-sectional diameter in the range of 2.2-2.5 nm and the pore diameter of 3.7 nm, another possible explanation is that a second molecule can try to enter the pore during the sojourn of the first molecule inside the pore. In this scenario, deeper blockades will occur at random times depending on the arrival frequency of the second molecule as well as the lingering time of the second molecule not being able to penetrate into the pore because there is already the first molecule inside the pore. While the voltage dependence of fraction of deeper events in Supplementary Figure 1 can be rationalized with this scenario, this cannot explain the duration of the deeper blockade events decreasing with voltage as in Figure 3f and 5d. Thus, we interpret the deep blockages as chain folding.

This interpretation is corroborated by analysis of the multi-level steps in deep blockages. Using the OpenNanopore software developed by Raillon et al. [3] to get amplitude and duration of each level in multi-level events, we plotted the accumulated duration *versus* I_b/I_0 at each voltage by using our custom MATLAB code. Aside from the absence of a detectable intra-pore-double-structured population and an increase in the fraction of moderate blockages, these plots show similar distributions as I_b/I_0 histograms given in Figure 3c and Supplementary Figure 3, which were constructed using the MATLAB code written by Plesa and Dekker [4] and OriginPro (OriginLab Corporation, USA). An example (120 mV) is given in Supplementary Figure 17. For further analysis, we grouped the current level in the deepest I_b/I_0 as level 2, the moderate level as level 1, and the shallowest level as level 0. Using our custom MATLAB code, we found that only 0.82 to 6.6% of deep blockages (events containing level 2) were single-level events, and 59 to 96% of deep blockages have more than

2 steps. Among four possibilities of combination of the first level group and the last level group (1/1, 2/1, 1/2, 2/2) for multi-level deep events, the case starting with moderate blockage step and ending with moderate blockage step (1/1) is the most probable at all voltages ranging from 42 to 82%. This preferred sequence of current levels in deep blockages supports our 'chain-folding' scenario since it is probable that single-file part of chain is inserted to the pore entrance first and then chain folds and unfolds within the pore during the translocation until single-file tail of chain finally goes through the narrow pore.

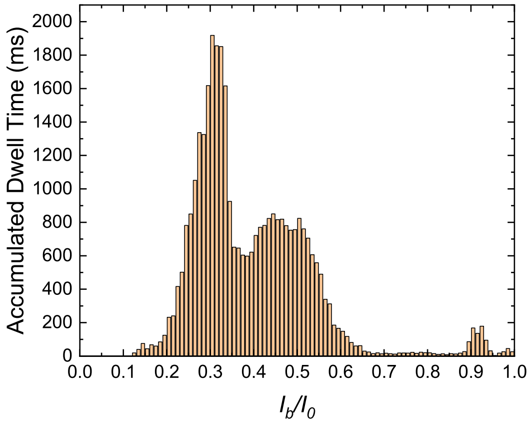

Supplementary Fig. 22: Accumulated dwell time of blockage steps *versus* blockage current ratio (I_b/I_0) at 120 mV. The data in Figure 3 in our revised manuscript was re-analyzed by using OpenNanopore software [3] and our custom MATLAB code.

Supplementary Note 2

Charge regulation by counterion binding: The dissociation constant K for the ionic equilibrium between each of the monopoles of the zwitterion and its corresponding counterion, $C^+A^- \rightarrow C^+ \; + \; A^-$ (where C^+ denotes a cation, as the quaternary ammonium group in Figure 1a, and A^- denotes an anion, as the Cl^-) is well known as described in [5, 6]

$$K = \frac{[C^+][A^-]}{[C^+A^-]} = \frac{\alpha}{1-\alpha}[A^-] = \exp(\frac{\Delta G}{k_B T}), \tag{1}$$

where α is the effective degree of ionization after charge regulation by counterion binding, the square brackets indicate the activities, ΔG is the free energy of formation of the ion-pair from the dissociated ions, and $k_B T$ is the Boltzmann constant times the temperature. Using the expression for ΔG and the above equation, the effective degree of ionization is connected to the local dielectric constant ϵ_ℓ as

$$\alpha = \left(1 + [A^-]\exp(\frac{e^2}{4\pi\bar{\epsilon}\epsilon_\ell k_B T r})\right)^{-1}, \tag{2}$$

where $[A^-]$ is the activity of the counterion and r is the distance of the counterion from the ionic group. The above relation gives the connection between the local dielectric constant and the differential counterion binding of the monopoles resulting in CSB in polyzwitterions. Furthermore, α (equivalently the effective charge) of the ionic groups in the zwitterion determines the translocation time of polyzwitterion molecules. The relation between the charges of the ionic groups and the translocation time is derived below, enabling inference of the magnitude of CSB from translocation experiments.

Supplementary Note 3:

Model for translocation kinetics: The generic scenario of translocation of a single chain of length N (in units of monomer length ℓ) through a narrow nanopore of length M (in units of ℓ) is sketched in Figure 6a, where the chain moves from the donor chamber (left) to the receiver chamber (right) under an external electric field $\vec{E}$. This process involves three stages involving four states. In the first stage, upon capture of the chain at the pore mouth (state 1, with N monomers in the donor chamber), the chain is squeezed into the nanopore until the pore is filled (state 2, with M monomers in the pore and $N - M$ monomers in the donor chamber by negotiating an entropic barrier). In the second stage, $N - M$ monomers are translocated into the receiver chamber (state 3, with M monomers in the pore and $N - M$ monomers in the receiver chamber). In the third stage, the pore is depleted from state 3 to state 4 (with N monomers in the receiver chamber side). The free energy landscape (free energy F versus the translocation coordinate m, defined below) associated with these three stages is sketched in Figure 6b.

As sketched in Figure 6c, the nanopore is a uniform cylinder of length M in units of monomer length ℓ, axially centered along the x-axis (in units of ℓ), with $x = 0$ at the pore entrance. The voltage drop across the nanopore is ΔV such that the electric field is in the negative x-direction with a constant strength $E = \Delta V/M$. The electric potential $\psi(x)$ at the location x inside the pore is $\Delta V x/M$. The number of zwitterion monomers per chain is N. When the polyzwitterion monomers are inside the nanopore, the chain backbone is aligned along the x-axis, and pendant groups are distributed uniformly about the chain backbone axis. Each pendant zwitterionic group inside the pore subtends an angle θ to the backbone with the monopole charges q_1 and q_2, and the charge separation distance is d (in units of ℓ). The x-position of the center of the charge separation for the i-th zwitterionic group is x_i (Figure 6c). In the case of PSBMA, q_1 and q_2 denote positive and negative charges, respectively, and vice versa for PMPC.

Supplementary Note 4

Derivation of charge-electric field interaction (F_{pE}): Assuming that $\psi(x)$ at the normal plane at x is the same across the plane, the electrostatic contribution to the free energy due to the presence of the i-th monomer inside the pore is $F_{pE,i} = eq_1\psi(x_i - \frac{d}{2}\cos\theta) + eq_2\psi(x_i + \frac{d}{2}\cos\theta)$, given as

$$\frac{F_{pE,i}}{k_BT} = eq\psi(x_i) - e\mathbf{p}\cdot\mathbf{E}, \tag{3}$$

where $q = q_1 + q_2$ is the total charge, and $p = (q_1 - q_2)d/2$ is the magnitude of dipole moment (for PSBMA, Figure 6c). The first and second terms on the right-hand side of the above equation denote the monopole and dipole contributions, respectively. Since $\psi(x) = \Delta V x/M$, $F_{pE,i}$ is given by

$$\frac{F_{pE,i}}{k_BT} = \frac{v}{M}(qx_i - p'), \qquad v = e\frac{\Delta V}{k_BT}, \qquad p' = (q_1 - q_2)(\frac{d}{2}\cos\theta). \tag{4}$$

When the length of m monomers has entered the nanopore, the net free energy due to polymer-electric field interaction follows from Supplementary Equation (4) as

$$\frac{F_{pE}(m)}{k_BT} = \sum_1^m \frac{F_{pE,i}}{k_BT} = \int_0^m dx\frac{F_{pE}(x)}{k_BT} = \int_0^m dx\frac{v}{M}(qx - p') = \frac{v}{M}(q\frac{m^2}{2} - p'm). \tag{5}$$

$F_{pE}(m)$ for all three stages of translocation are given in Supplementary Table 1.

Supplementary Note 5

Contributions to free energy landscape: (a) Conformational entropy: The partition sum Z_N of a chain of N monomers with one end anchored at the pore entrance (state 1 in Figure 6a) is $z^N N^{\gamma-1}$ so that the Helmholtz free energy $F_{b,\text{ent}}(N)$ due to conformational entropy is given as,

$$\frac{F_{b,\text{ent}}(N)}{k_BT} = -N\ln z + (1-\gamma)\ln N, \tag{6}$$

where z is the coordination number in the bulk (taken as 4), and γ is the critical surface exponent. When m monomers are inside the nanopore, the number of conformations is z_p^m, where z_p is the number of states each monomer can assume inside the pore (taken as 3). Hence the Helmholtz free energy $F_{p,\text{ent}}(m)$ of this single file conformation of m monomers inside the nanopore is

$$\frac{F_{p,\text{ent}}(m)}{k_BT} = -m\ln z_p. \tag{7}$$

Using Supplementary Equations (6) and (7), the contribution from conformational entropy to the free energy F_{ent} for all three stages of translocation can be readily obtained and the results are given

in Supplementary Table S1.

(b) Pore wall-chain interaction: We parametrize the interaction between the monomers inside the pore and the pore wall with a constant ϵ_0 per monomer ($\epsilon_0 > 0$ for a repulsive pore wall). The free energy $F_{\text{pore}}(m)$ due to interaction of m monomers inside the pore with the pore surface is

$$\frac{F_{\text{pore}}(m)}{k_B T} = \epsilon_0 m, \tag{8}$$

as included in Supplementary Table S2.

(c) Free energy in receiver chamber: Since the voltage difference in the receiver chamber, compared to the donor chamber, is ΔV, the electric contribution to the free energy of a chain in the donor chamber is taken as zero, and that of a chain of m monomers in the receiver chamber is

$$\frac{F_b(m)}{k_B T} = q_b v m, \tag{9}$$

where q_b is the net charge of each of the zwitterion group in the receiver chamber. In this study, we have taken q_b as the same net charge $q_1 + q_2$ of the repeat unit inside the pore. $F_b(m)$ for stages 2 and 3 is included in Supplementary Table S2.

Supplementary Note 6

Approximation of ϵ_0 (interaction energy between one monomer and pore wall): We estimate the pore wall-polymer interaction energy per monomer (ϵ_0) by calculating the sum of electric energies of the two charged groups within a repeat unit using Debye-Hückel potential from the surface charge within an area of square of monomer length (ℓ^2).

Debye-Hückel potential at distance, R, from the charged surface is calculated by $\psi(R) = \frac{ez}{4\pi\epsilon_0\epsilon}\frac{e^{-\kappa R}}{R}$, where κ^{-1} is Debye length, ez is charge of the surface area of ℓ^2, e is the elementary charge, ϵ_0 is the permittivity of vacuum, and ϵ is the static dielectric constant of medium. For bulk water, we use 80 for ϵ. For ez, from a previous work [1], we set -0.12 C/m^2 as the surface charge density of silicon nitride pore submerged in 1 M KCl at pH 7. With this surface charge density, a segment of pore surface with area of ℓ^2 has a surface charge (ez) of -0.047e with 2.5 nm used for ℓ. κ is estimated as 1.2ℓ by $\kappa^{-1} \sim \frac{0.3}{c_s} nm$, where c_s is salt concentration (1 M). Using estimated distance to the pore surface from the two charged groups (5.1ℓ for q_1 and 3.0ℓ for q_2), the potential for q_1 (ψ_1) is calculated to be -9.2×10^{-6} V and that for q_2 (ψ_2) is calculated to be -9.3×10^{-5} V.

The sum of electrostatic interaction energy with ez of q_1 and q_2 (ϵ_0) is calculated by $\frac{1}{k_B T}\sum_i q_i e \psi_i$. By fixing q_2 as -0.25 and varying q_1 from +0.20 to +0.05, the range of ϵ_0 is calculated to be from

0.00086 to 0.00091. Thus, we use 0.0009 for ϵ_0 in our Mathematica code. We note that the value is so small that its effect is negligible in calculated free energy profiles and translocation times.

Supplementary Note 7

Mean translocation time: Assuming that the translocation time is much longer than the conformational relaxation time of the polymer in the donor chamber, which is valid in this system, and using the Fokker-Planck formalism for the drift-diffusion process of translocation, the probability $P(m,t)$ that m monomers have translocated at time duration t is given by [7, 8]

$$\frac{\partial P(m,t)}{\partial t} = k_0 \left[\frac{\partial}{\partial m} \left(\frac{1}{k_B T} \frac{\partial F(m)}{\partial m} P(m,t) \right) + \frac{\partial^2 P(m,t)}{\partial m^2} \right], \tag{10}$$

where k_0 is the diffusion coefficient of a monomer inside the nanopore assumed to be the same for all monomers. Using the reflecting boundary condition at the pore entrance and the absorbing boundary condition at the pore exit, the mean translocation times follow from Supplementary Equation (10) as [7, 8]

$$\langle \tau \rangle = \frac{1}{k_0} \int_0^{N+M} dy \; e^{F(y)/k_B T} \int_0^y dz \; e^{-F(z)/k_B T}, \tag{11}$$

where $F(y)$ is given in equation (2).

Supplementary Note 8

Hydrolysis of PSBMA: The carboxylic esters in SBMA can be hydrolyzed to acrylic acid in water, and a previous work [9] showed around 12% hydrolysis of their PSBMAs. However, our sample of PSBMA was synthesized in 2, 2, 2-trifluoroethanol (TFE) (and not in water), where hydrolysis of monomers was prevented. Furthermore, pH titration of our PSBMA solution with HCl from pH 7 to around pH 2.5 showed no buffering range as in Supplementary Figure 1a, which means that the PSBMA solution had no basic moieties to be titrated. We also inspected ^{1}H NMR spectrum of PSBMA in D_2O, which showed no detection of hydrolysis as in Supplementary Figure 1b.

References

[1] Lin, K. et al. Surface charge density inside a silicon nitride nanopore. *Langmuir* **37,** 10521-10528 (2021).

[2] Lin, C.-Y. et al. Modulation of charge density and charge polarity of nanopore wall by salt gradient and voltage. *ACS Nano* **13,** 9868-9879 (2019).

[3] Raillon, C., Granjon, P., Graf, M., Steinbock, L. J., & Radenovic, A. Fast and automatic processing of multi-level events in nanopore translocation experiments. *Nanoscale* **4,** 4916-4924 (2012).

[4] Plesa, C. & Dekker, C. Data analysis methods for solid-state nanopores. *Nanotechnology* **26,** 084003 (2015).

[5] M. Daune, *Molecular Biophysics: Structures in motion* (Oxford University Press, Oxford, 2003).

[6] Robinson, R. A. & Stokes, R. H. *Electroyte Solutions* (Dover, 2002).

[7] Muthukumar, M. *Polymer Translocation* (CRC Press, Boca Raton, 2011).

[8] Risken, H., *The Fokker-Planck Equation* (Springer, Berlin, 1989).

[9] Mary, P., Bendejacq, D. D., Labeau, M. P. & Dupuis, P. Reconciling low- and high-salt solution behavior of sulfobetaine polyzwitterions. *J. Phys. Chem. B.* **111,** 7767–7777 (2007).
